# Ten Years after the International Committee of Medical Journal Editors’ Clinical Trial Registration Initiative, One Quarter of Phase 3 Pediatric Epilepsy Clinical Trials Still Remain Unpublished: A Cross Sectional Analysis

**DOI:** 10.1371/journal.pone.0144973

**Published:** 2016-01-06

**Authors:** Anette Lampert, Georg F. Hoffmann, Markus Ries

**Affiliations:** 1 Department of Clinical Pharmacology and Pharmacoepidemiology, University of Heidelberg, Heidelberg, Germany; 2 Cooperation Unit Clinical Pharmacy, University of Heidelberg, Heidelberg, Germany; 3 Pediatric Neurology and Metabolic Medicine, Center for Rare Disorders, Center for Pediatric and Adolescent Medicine, Heidelberg University Hospital, Heidelberg, Germany; Mario Negri Institute for Pharmacology Research, ITALY

## Abstract

**Introduction:**

Although selective reporting of clinical trial results introduces bias into evidence based clinical decision making, publication bias in pediatric epilepsy is unknown today. Since there is a considerable ambiguity in the treatment of an important and common clinical problem, pediatric seizures, we assessed the public availability of results of phase 3 clinical trials that evaluated treatments of seizures in children and adolescents as a surrogate for the extent of publication bias in pediatric epilepsy.

**Methods:**

We determined the proportion of published and unpublished study results of phase 3 clinical trials that were registered as completed on ClinicalTrials.gov. We searched ClinicalTrials.gov, PubMed, and Google Scholar for publications and contacted principal investigators or sponsors. The analysis was performed according to STROBE criteria.

**Results:**

Considering studies that were completed before 2014 (N = 99), 75 (76%) pediatric phase 3 clinical trials were published but 24 (24%) remained unpublished. The unpublished studies concealed evidence from 4,437 patients. Mean time-to-publication was 25 SD ± 15.6 months, more than twice as long as mandated.

**Conclusion:**

Ten years after the ICMJE’s clinical trials registration initiative there is still a considerable amount of selective reporting and delay of publication that potentially distorts the body of evidence in the treatment of pediatric seizures.

## Introduction

*“In the nineteenth century health was transformed by clear*, *clean water*. *In the twenty-first century*, *health will be transformed by clean clear knowledge*.*”*

Sir Muir Gray

It is estimated that 30–50% of all the clinical trials that have been conducted and completed are still not published in academic journals [1–5]. However, publication bias in pediatric epilepsy has not been investigated. Epilepsy is a relatively frequent, serious condition associated with high morbidity for patients [6]. Pediatric seizures can have a deleterious impact on a child’s development causing disability and lifelong dependency [7]. In addition, seizures, hospitalizations, emergency department visits, or medication burden disrupt lives of patients and afflicted families [7]. Clinically available anti-epileptic drugs fail to control seizures in approximately 30% of epileptic patients [8, 9]. Beyond pharmacoresistance the long-term use of anti-epileptic drugs is limited by adverse events, drug-drug-interactions, and non-compliance due to inconvenient regimens [10–12].

Pediatric treatment decisions are often based on incomplete clinical data and are characterized by off-label use due to lack of clinical trials in children [13]. Waste of knowledge due to incomplete publication of trial results impedes complete assessment of the effect of an intervention [14]. Indeed, outcome data that favor the efficacy of the investigated drug are twice as likely to be published [1, 15, 16]. Consequently, when unfavorable results of drug trials are not published, the efficacy of a drug may be overestimated and trials may be unnecessarily repeated which consequently wastes resources. Considering insufficient clinical data in pediatrics, publication bias can particularly distort the apparent efficacy of a drug which complicates the interpretation of medical literature and decision making about an individual treatment [15, 17, 18]. Of note, several historical examples demonstrate that retention of findings especially concerning adverse events seriously impairs treatment decisions [19–21]. For example, the retention of reporting increased mortality rates during clinical trials with the antiarrhythmic drug lorcainide in 1980, concealed early warnings regarding the risk for cardiac death [21,22]. Beyond the impact on treatment decisions which affects all patients, there is an explicit ethical obligation to publish towards study participants mandated by the Declaration of Helsinki. Patients participate in clinical research on the understanding that findings will be of public interest. Therefore, non-publication of trial outcome data violates an ethical obligation that investigators have towards study participants [23,24].

Phase 3 clinical trials are usually rigorously designed, comparing the investigational compound against placebo or an active control in the population of interest and thus, provide a high level of evidence [25]. In addition, systematic reviews and meta-analysis investigating treatment options and treatment guidelines are largely based on such trials [17]. Therefore, publication bias of phase 3 clinical trials can have a pronounced impact on therapeutic decisions. Since the current publication bias in phase 3 clinical trials in children and adolescents with epilepsy is unknown, we assessed the public availability of phase 3 clinical trials addressing treatments of seizures in children and adolescents as a surrogate for the extent of publication bias in pediatric epilepsy. This analysis aimed at highlighting the current publication bias facilitating more cautious interpretation of current knowledge in pediatric epilepsy treatment decisions.

## Methods

The analysis was performed according to STROBE (STrengthening the Reporting of OBservational studies in Epidemiology) criteria.

### ClinicalTrial.gov query

For cross sectional analysis, we searched ClinicalTrials.gov on February 3, 2015 with the key word ‘epilepsy’ and restricted search to completed phase 3 clinical trials with children. ClinicalTrials.gov defines the recruiting status as completed when *“the clinical study has ended normally*, *and participants are no longer being examined or treated (i*.*e*, *the "last subject*, *last visit" has occurred)”*. The search output was downloaded in a spreadsheet format representing the basis for analysis. For the analysis all completed phase 3 clinical trials were included that were registered on ClinicalTrials.gov investigating the treatment of pediatric seizures, irrespective of the interventions studied (e.g., surgical procedures or drugs). In addition, terminated phase 3 clinical trials with children investigating treatments for epilepsy were analyzed for publication status and reasons for termination provided on ClinicalTrials.gov. ClinicalTrials.gov defines the recruiting status as terminated when *“the clinical study has stopped recruiting or enrolling participants early and will not start again*. *Participants are no longer being examined or treated*. As such, a terminated trial does not represent a fully completed data collection available for publication and thus, terminated trials were analyzed separately. A trial was considered as published when results were presented in the ClinicalTrials.gov record or when a journal had published a peer-reviewed manuscript online or in print, which included any outcome data from the trial in question. In a sensitivity analysis, we took an additional conservative approach to the publication expectation and assessed trials completed before 2014 separately in accordance with the Food and Drug Administration Amendments Act (FDAAA) of 2007 that requires publication within one year after completion of the trial [26].

### Publication search

When the ClinicalTrial.gov record did not provide results we searched for manuscripts by reviewing the ClinicalTrials.gov record for references to published outcome data. ClinicalTrial.gov encourages investigators to provide a link to PubMed indexed manuscripts that contain results from the registered trial. In addition, ClinicalTrial.gov uses the unique trial identification number (NCT number) to automatically identify and link corresponding PubMed entries. For trials not providing a corresponding PubMed link we searched PubMed and Google Scholar for the trials in question. Search terms for PubMed and Google Scholar included the NCT number, other study ID numbers listed in the ClinicalTrials.gov record, the investigated drug, ‘epilepsy’ or the specified condition, and details of the study design (e.g., ‘double blind’, ‘randomized’, or inclusion and exclusion criteria). Registry entries and publications identified by the PubMed and Google Scholar search were matched by consensus based on the following study characteristics: study title, investigated drug, study design, number of participants, and inclusion and exclusion criteria. If no published results of registered trials could be identified, the principal investigator or sponsor listed in the ClinicalTrials.gov record was contacted.

#### Time-to-publication

We calculated the time-to-publication as the number of months from the primary completion date of the trial and the publication of results either on ClinicalTrials.gov or in a peer-reviewed Journal. ClinicalTrials.gov defines the primary completion date as *“the date that the last participant in a clinical study was examined or received an intervention and that data for the primary outcome measure were collected*.*[…] The primary completion date is the term used on ClinicalTrials*.*gov for "completion date" defined in Section 801 of the Food and Drug Administration Amendments Act of 2007”*. If the primary completion date was not available the completion date of the trial was used. The calculation of time-to-publication was limited to studies completed before 2014 in accordance with the FDAAA that requires publication within one year after completion of the trial [26].

### Statistical analysis

The following continuous or categorical variables were analyzed: NCT number, study title, gender, age, study phase, study type, study design, condition, intervention, recruitment status, primary completion date and completion date, availability of study results, publication date, time-to-publication, sponsor, and funding source. Standard methods of descriptive statistics were applied. All calculations were performed with SAS Enterprise Guide version 5.1 (SAS, Cary, NC, USA).Two-sided p values ≤0.05 were considered statistically significant.

## Results

### Pediatric phase 3 clinical trials investigating antiepileptic treatment

**Fig 1:**
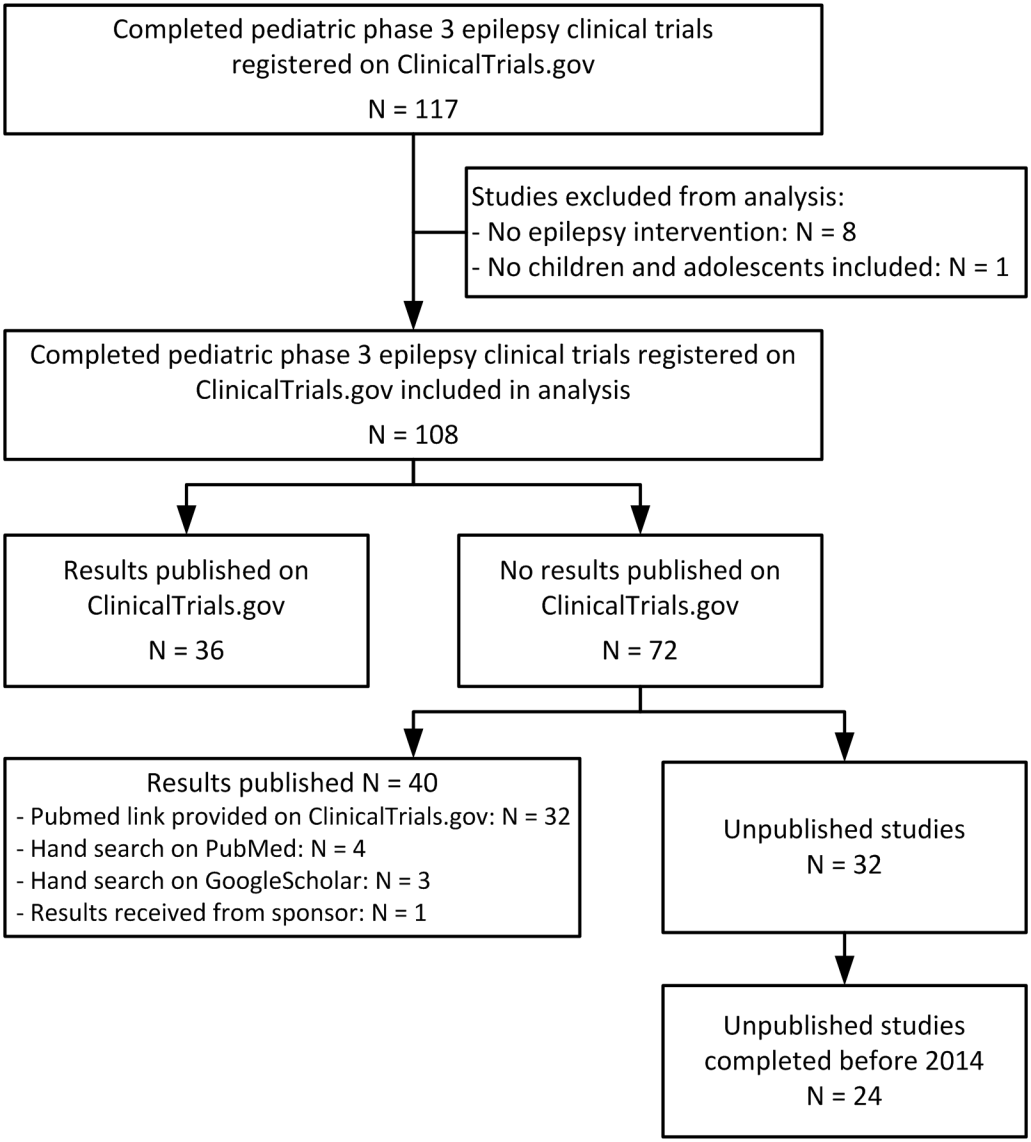
Identification of published and unpublished pediatric phase 3 epilepsy clinical trials registered on ClinicalTrials.gov: study flow diagram.

**Fig 2:**
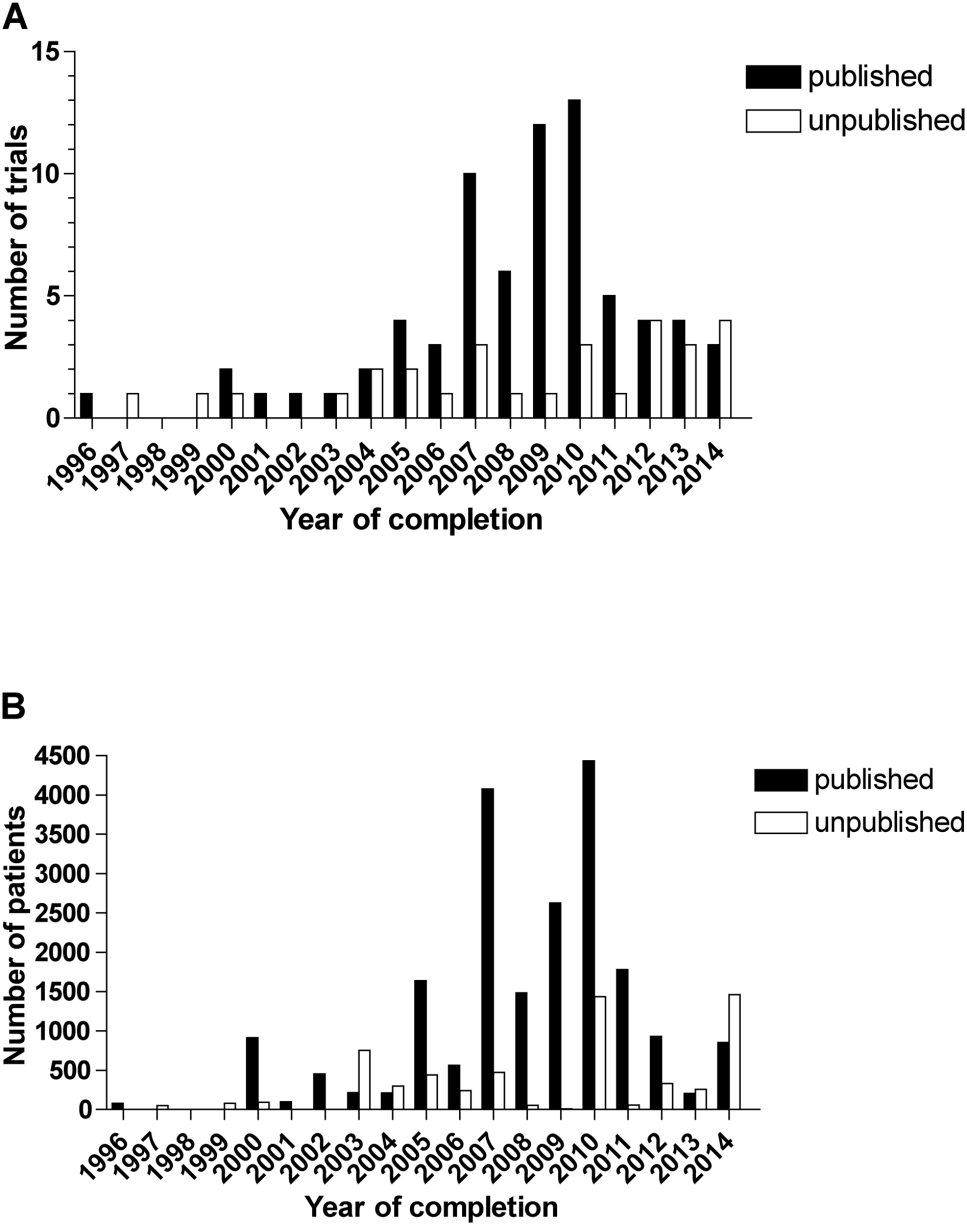
Published and unpublished pediatric phase 3 epilepsy clinical trials. A) Number of trials by year of completion. B) Number of enrolled patients by year of completion.

The ClincalTrials.gov search initially identified 117 completed trials (Fig 1). We subsequently excluded eight trials from the analysis because they did not investigate epilepsy and one trial because no children or adolescents were enrolled. Therefore, a total of 108 completed phase 3 clinical trials investigating anti-epileptic treatment in children and adolescents registered on ClinicalTrials.gov were searched for publication of results. The year of completion ranged from 1996 to 2014 (Fig 2). The completion date of six studies remained unknown whereof three studies were published in 2007 and 2009 and thus, considered for analysis as completed before 2013. A total of 15 terminated phase 3 pediatric epilepsy clinical trials were identified (Table 1). Reasons for termination were multifaceted (e.g., recruitment challenges, unlikely attaining a positive outcome, or Sponsor’s decision).

**Table 1:** Terminated phase 3 pediatric epilepsy clinical trials and reasons for termination.

Study title and ClinicalTrials.gov Identifier	Investigated compound	Reason for termination
An Open-label Pilot Study Using Carvedilol-CR as a P-glycoprotein Inhibitor as Adjunct Therapy in the Treatment of Medically-refractory Epilepsy (NCT00524134)	carvedilol	Principal investigator left the institution
A Double-blind, Randomised, Placebo-controlled, Multi-centre Study to Assess the Efficacy and Safety of Adjunctive Zonisamide in Myoclonic Seizures Associated With Idiopathic Generalised Epilepsy* (NCT00693017)	zonisamide	Sponsor's decision
A Randomized, Double-Blind, Parallel-Group, Multicenter Study to Evaluate the Retention Rate, Efficacy, Safety, and Tolerability of Carisbamate, Topiramate and Levetiracetam as Adjunctive Therapy in Subjects With Partial Onset Seizures (NCT00563459)	carisbamate, topiramate, levetiracetam	Carisbamate partial onset seizures studies lacked consistent efficacy data so trials in this indication were terminated
HEAD-TO-HEAD Evaluation of the Antiepileptic Drugs Levetiracetam (LEV) vs. Sulthiame (STM) in a German Multi-Centre, Doubleblind Controlled Trial in Children With Benign Epilepsy With Centro-Temporal Spikes (NCT00471744)	levetiracetam, sulthiame	Low patient number after 2 years recruiting
An International, Double-blind, Randomized, Multi-center, Parallel Group, Historical-control Conversion to Monotherapy Study to Evaluate the Efficacy and Safety of Brivaracetam in Subjects (≥ 16 to 75 Years Old) With Partial Onset Seizures With or Without Secondary Generalization (NCT00699283)	brivaracetam	An interim analysis revealed the study was unlikely to attain a positive outcome for the efficacy analysis. No safety concerns were detected
An International, Double-blind, Randomized, Multi-center, Parallel Group, Historical-control Conversion to Monotherapy Study to Evaluate the Efficacy and Safety of Brivaracetam in Subjects (≥ 16 to 75 Years Old) With Partial Onset Seizures With or Without Secondary Generalization (NCT00698581)	brivaracetam	An interim analysis revealed the study was unlikely to attain a positive outcome for the efficacy analysis. No safety concerns were detected
A Double-blind, Randomised, Placebo-controlled Multi-centre Study to Assess the Efficacy and Safety of Adjunctive Zonisamide in Primary Generalised Tonic Clonic Seizures* (NCT00692003)	zonisamide	Sponsor's decision
RTG113388, a Long-term, Open-label Safety Extension Study of Retigabine/Ezogabine in Pediatric Subjects With Partial Onset Seizures (≥ 12 Years Old) and Subjects With Lennox-Gastaut Syndrome (≥12 Years Old)* NCT01668654	retigabine/ ezogabine	FDA placed a clinical hold on the Pediatric Program requiring retigabine discontinuation in subjects; early termination allows for timely reporting of results
An Open-Label Safety Study of USL261 in the Outpatient Treatment of Adolescent and Adult Subjects With Seizure Clusters (NCT02161185)	intranasal midazolam	Study terminated due to slow enrollment. There were no safety concerns.
A Study of the Safety and Efficacy of Depakote Sprinkle Capsules in the Treatment of Partial Seizures in Children (NCT00067431)	divalproex sodium	not provided
A Double-Blind Study to Evaluate the Effectiveness and Safety of RWJ-333369 as Adjunctive Therapy in Korean and Japanese Patients With Partial Onset Seizures (NCT00697762)	carisbamate	The trial was stopped based on information from the global phase 3 studies.
Multi-site, Prospective, Open-label, Long-term, Flexible Dose, Interventional Study to Evaluate the Safety and Tolerability of Clobazam as Adjunctive Therapy in Paediatric Patients Aged ≥1 to ≤16 Years With Dravet Syndrome (NCT02187809)	clobazam	The study was terminated due to recruitment challenges
Carbon Dioxide (Carbogen) for the Treatment of Febrile Seizures (NCT01370044)	carbogen	results of interim analysis (not safety relevant)
Buccal, Intranasal or Intravenous Lorazepam for the Treatment of Acute Convulsions in Children in Blantyre, Malawi: a Randomized Trial (NCT00343096)	lorazepam	The buccal arm of the study was 30% less effective in stopping seizures within 10 minutes compared with the IV dose. This met a stopping rule for the study
A Randomized Clinical Trial for the Treatment of Refractory Status Epilepticus* (NCT00265616)	propofol, thiopental/ pentobarbital	Insufficient recruitment

*results provided on ClinicalTrials.gov

### Publication status—trials, participants, and time-to-publication

**Fig 3:**
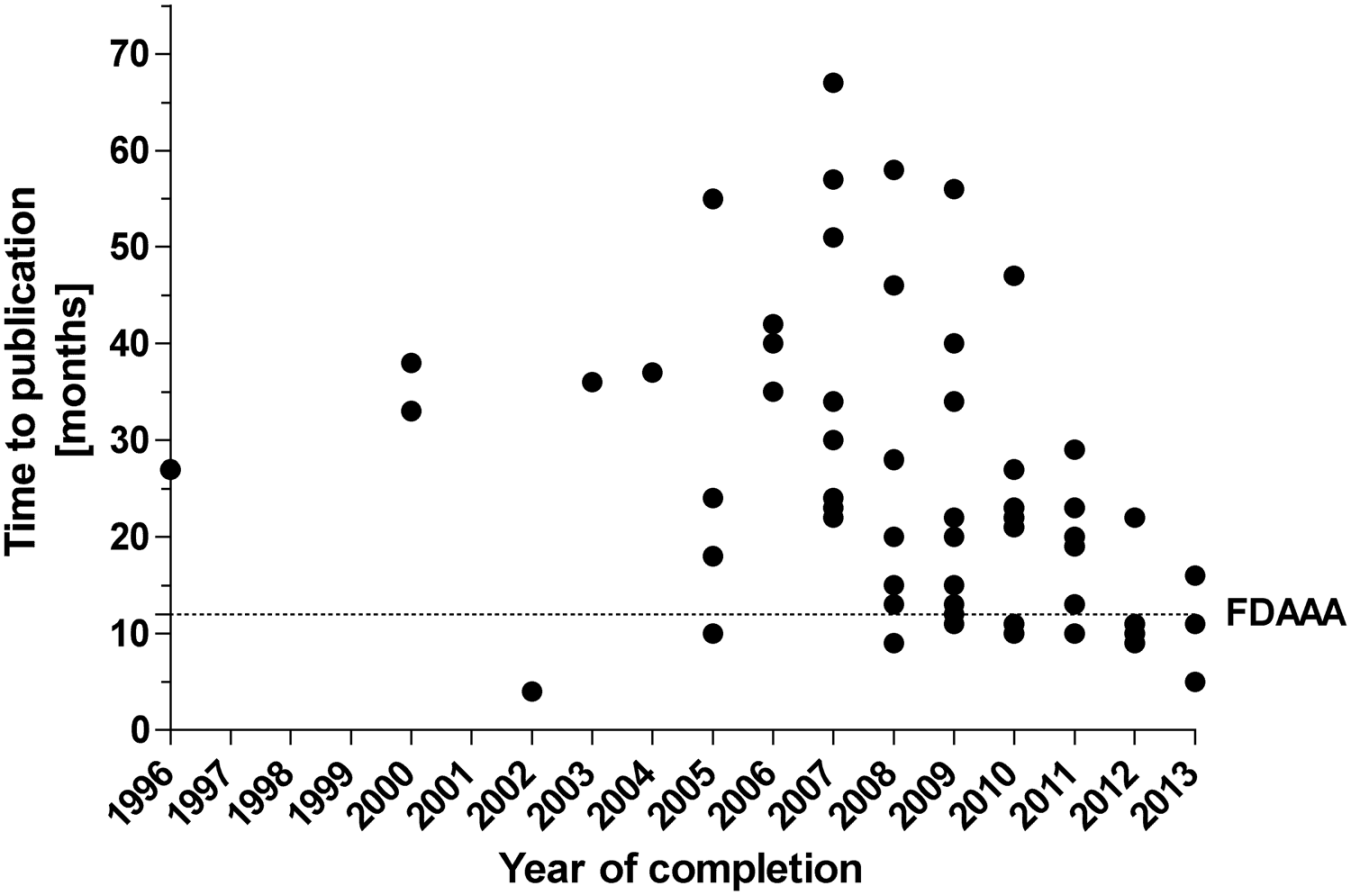
Time-to-publication of pediatric phase 3 epilepsy clinical trials (completed before 2014). “FDAAA” indicates the timeline mandated by the Food and Drug Administration Amendments Act of 2007.

Overall, 76 (70%) completed studies were published and 32 (30%) completed studies have remained unpublished. Considering only studies that were completed before 2014 (N = 99), which was the current deadline mandated by the FDAAA, that requires publication of results within one year after completion of the trial, 75 (76%) completed studies were published and 24 (24%) remained unpublished. A total of 27,587 patients were enrolled in the identified completed studies (N = 107 studies with available data) (Table 2). Up to date, data involving 6,464 participants are not yet publically available and 4,437 participants when considering studies completed before 2014. The mean time-to-publication, i.e. the delay from completion of the trial until public availability of the outcome data, was 25 SD ± 15.6 months (median 22, IQR 11 to 34, N = 70 trials with available data) for studies that were completed before 2014 (Fig 3). Overall 33% (23/70) studies were published within a year as mandated by FDAAA. Considering published studies, time-to-publication showed a decreasing trend over the years. When considering the mean publication time of 25 months and thus analyzing studies completed before 2013 (N = 92), 71 (77%) studies were published and 21 (23%) remained unpublished. Terminated trials provided results for four studies on clinicaltrials.gov.

**Table 2:** Number of patients enrolled in completed pediatric phase 3 epilepsy clinical trials.

	Number of patients enrolled (N = 27,587)	Median size of the trial (IQR)
Published trials	21,123 (77%) (N = 75 trials with available data)	207 (IQR 97 to 298)
	*20,575 (82%)(N = 74 trials with available data	*206 (IQR 98 to 396)
Unpublished trials	6,464 (23%)(N = 32 trials with available data)	97.5 (IQR 45 to 286)
	*4,437 (18%) (N = 24 trials with available data)	*85 (IQR 51 to 204)

*Trial was completed before 2014 (which takes into account the FDAAA deadline to publication of 1 year after completion). FDAAA = Food and Drug Administration Amendments Act of 2007

### Compounds investigated, demography, and funding

The identified completed pediatric phase 3 clinical trials investigated various anti-epileptic interventions (Table 3). Thereby, the most investigated anti-epileptic treatment was levetiracetam with 17 published trials and six unpublished trials. Studies investigating brivaracetam remained more often unpublished than published. Brivaracetam, lecetiracetam, and topiramate had more than three unpublished studies. The majority of the identified pediatric trials included both genders, and had enrolled also adults and elderly patients in addition to children (Table 4). Twenty-one percent (5/23) trials including solely children remained unpublished. The majority of identified trials were funded by pharmaceutical industry.

**Table 3:** Compounds or interventions investigated in registered pediatric phase 3 epilepsy clinical trials.

Compound/Intervention	Published trials (N)	Unpublished trials (N)
Brivaracetam	3	5*
Carbamazepine	2	0
Carisbamate	3	3
Clobazam	2	0
Diagnostic procedure (MEG vs. PET vs. MRI)	0	1
Diazepam	2	0
Eslicarbazepine	0	2
Fosphenytoin	0	1
Gabapentin	2	0
Lacosamide	9	3**
Lamotrigine	9	0
Levetiracetam	17	6*
Lorazepam	4	0
Melatonin	0	1
Midazolam	1	0
Modified Atkins diet	2	0
Natural progesterone	1	0
Oxcarbazepine	2	2*
Paraldehyd	1	0
Perampanel	3	0
Phenytoin	1	1
Prednisolone	1	0
Pregabalin	3	0
Procedure (surgery)	1	0
Rufinamide	3	1
Topiramate	7	5
Valproate	2	1
Zonisamide	4	1

Ten studies investigated multiple compounds.

*One trial or **two trials were completed in 2014.

MEG = magnetoencephalography, PET = positron emission tomography, MRI = magnetic resonance imaging

**Table 4:** Demographic data of study population and funding source of registered pediatric phase 3 epilepsy clinical trials.

	Published trials (N)	Unpublished trials (N)
Gender		
Male	0	0
Female	1	0
Both	75	32
Age group		
Child	18	5
Child/Adult	14	4
Child/Adult/Elderly	44	23
Funding source		
Industry	65	27
NIH	1	0
NIH/Other*	1	2
Other*	9	3

*Other: e.g., other government or academic institutions, or hospitals; NIH = National Institutes of Health

## Discussion

Almost every fourth completed phase 3 clinical trial that investigates anti-epileptic interventions in children and adolescents remains unpublished. Consequently, results from 4,437 study participants are not available for clinical decision-making when considering unpublished trials completed before 2014. This induces a considerable publication bias into the assessment of efficacy and safety of interventions intended to treat pediatric seizures. As a first measure to address publication bias, ten years ago the International Committee of Medical Journal Editors (ICMJE) has required that prospective trials involving human participants must be registered prior to the beginning of study enrollment in order to be considered for publication in member journals [27]. This requirement was later incorporated into the CONSORT statement for reporting clinical trials [28]. However, although the ICMJE requires trial registration as a precondition for publication, every fourth published trial is still not registered [29]. Therefore, there might be an unknown number of clinical trials that have been conducted without registration and consequently without publication of the results. The mean time-to-publication of the anti-epileptic pediatric phase 3 trials in the present study was 25 months which is more than twice as long as the time mandated by the FDAAA. In 2007 the prospective registration and mandatory publication of applicable trials (i.e., other than phase 1 clinical investigations, drugs with FDA approval, and at least one study site in the United States) within one year of completion became federal law in the United States with the FDAAA [26]. However, most trials do not comply with mandatory timely reporting [30]. In addition, this law is restricted to trials conducted in the US and requires only publication of trials that are completed after 2008. However, health care providers rely most of the time on evidence from trials done earlier to make treatment decisions. In particular, most of the anti-epileptic drugs used in pediatrics were approved several years ago, and thus, the rates of missing data may have a detrimental impact on current clinical practice when treating pediatric seizures. Therefore, the AllTrials initiative (www.alltrials.net) again calls for registration and publication of all results of all clinical trials—past and future—on all treatments in current use. Of interest, the publication pattern between published and unpublished reports was similar for all completed studies at any year and studies completed at certain cut-off years in a sensitivity analysis considering a) studies completed before 2014 (taking into account the FDAAA timeline of one year), and b) studies completed before 2013 (taking into account the mean time to publication of 25 months). Studies investigating treatments for epilepsy in children are particularly confronted with recruitment challenges leading to premature termination of studies. Recruitment challenges are a well-known barrier to pediatric clinical trials in general and thus, improving research infrastructure and consequent registration of studies to inform practitioners are needed to compound the problem [31]. Particularly pediatric societies should encourage members to improve the rate of independent studies and transparency of results and also to follow-up the benefit of approved drugs in pediatrics. There are no excuses for not publishing trial results because a low-threshold way to get any results published whether they are positive or negative is posting on a clinical trial registry, such as ClinicalTrials.gov. Researchers might fear to not getting the data published afterwards in a peer-reviewed journal. However, the ICMJE even encourages publication of clinical trial results in clinical trial registries and does not consider the posting of results as a barrier to publication in a member journal [32]. Terminated trials remained most likely unpublished due to incomplete data collection. Of interest, most phase 3 clinical trials involving children and adolescents with epilepsy are sponsored by the industry, therefore it is not surprising that most unpublished studies are industry-sponsored, too. This is nevertheless important taking into account the recent findings revealing that industry complies better with results reporting than NIH or other government or academic institution [33].

This study has several limitations. Since ClinicalTrials.gov is considered the most relevant clinical trial registry we did not investigate other databases (e.g., the German Clinical Trials Register or the Australian New Zealand Clinical Trials Registry). In addition, the investigation of a clinical trial registry implies that only registered trials were included in our analysis. In order to prevent misclassifying a trial as unpublished, we conducted an exhaustive literature search in two major databases (i.e., PubMed and GoogleScholar) with multiple search terms and contacted investigators or sponsors. Our study did not formally assess whether the content of the publications was consistent with the original research questions and the pre-specified statistical analysis plan since this information was not completely publically available for all trials. Furthermore, we did not formally monitor study sites or study sponsors whether the reported information in the public domain was correct. This analysis assumes that the entries provided on ClinicalTrials.gov are accurate and complete as mandated by the FDAAA [26]. Although the ClinicalTrials.gov allows examination of various aspects of ongoing and completed clinical trials, its ultimate usefulness depends on the research community to submit accurate, informative data.

Our data define the current publication bias in phase 3 clinical trials investigating anti-epileptic interventions ten years after the ICMJEs’ clinical trial registration initiative and represent today’s baseline for the future. Regular follow-ups will be of interest in order to document the impact of legal requirements and initiatives such as the AllTrials initiative on investigator’s compliance with publication of clinical trial outcome data. We hope that the publication efforts will increase over time in order to allow key-stakeholders more informed decisions based on true evidence for the benefit of children and adolescents with epilepsy.
